# Hyperglycemia: GDNF-EGR1 Pathway Target Renal Epithelial Cell Migration and Apoptosis in Diabetic Renal Embryopathy

**DOI:** 10.1371/journal.pone.0056731

**Published:** 2013-02-28

**Authors:** Ching-Yuang Lin, Tze-Yi Lin, Min-Chun Lee, Shih-Chieh Chen, Jeng-Shou Chang

**Affiliations:** 1 Clinical Immunology Center, China Medical University Hospital, Taichung, Taiwan; 2 Department of Pathology, China Medical University Hospital, Taichung, Taiwan; 3 College of Medicine, China Medical University, Taichung, Taiwan; 4 Department of Pediatrics, Buddhist Tzu Chi General Hospital, Taichung, Taiwan; University of Pecs Medical School, Hungary

## Abstract

Maternal hyperglycemia can inhibit morphogenesis of ureteric bud branching, Glial cell line-derived neurotrophilic factor (GDNF) is a key regulator of the initiation of ureteric branching. Early growth response gene-1 (EGR-1) is an immediate early gene. Preliminary study found EGR-1 persistently expressed with GDNF in hyperglycemic environment. To evaluate the potential relationship of hyperglycemia-GDNF-EGR-1 pathway, *in vitro* human renal proximal tubular epithelial (HRPTE) cells as target and *in vivo* streptozotocin-induced mice model were used. Our *in vivo* microarray, real time-PCR and confocal morphological observation confirmed apoptosis in hyperglycemia-induced fetal nephropathy via activation of the GDNF/MAPK/EGR-1 pathway at E12-E15. Detachment between ureteric branch and metanephrons, coupled with decreasing number and collapse of nephrons on Day 1 newborn mice indicate hyperglycemic environment suppress ureteric bud to invade metanephric rudiment. *In vitro* evidence proved that high glucose suppressed HRPTE cell migration and enhanced GDNF-EGR-1 pathway, inducing HRPTE cell apoptosis. Knockdown of EGR-1 by siRNA negated hyperglycemic suppressed GDNF-induced HRPTE cells. EGR-1 siRNA also reduced GDNF/EGR-1-induced cRaf/MEK/ERK phosphorylation by 80%. Our findings reveal a novel mechanism of GDNF/MAPK/EGR-1 activation playing a critical role in HRPTE cell migration, apoptosis and fetal hyperglycemic nephropathy.

## Introduction

Clinical and experimental evidence demonstrate that maternal diabetes induces a broad array of congenital malformation [1], [2], risk of birth defect in diabetic pregnancy ranging from two to six times above normal [3]. The developing kidney seems sensitive to high-glucose milieu [4]–[7]; with the fetus exposed to sustained high glucose ambience, damage affects multiple organs, a condition known as diabetic embryopathy [3], [8]. Animal study demonstrated that exposure to hyperglycemia *in utero* could cause nephron deficit in embryonic kidney development [9]. Decreased nephron numbers and inhibited morphogenesis of the ureteric bud branching in phase of metanephros development were well described in prior experiments [9], [10].

Organogenesis of a kidney proceeds in stages [11], [12]. Step 1 is formation of metanephric mesenchyme in intermediate mesoderm and subsequent outgrowth of the ureteric bud from Wolffian duct. Step 2 is invasion of ureteric bud into metanephric mesenchyme. Step 3 entails reciprocal inductive interaction between the ureteric bud and metanephric mesenchyme. Step 4 is further differentiation, including vascularization and inervation. Literature only identified interrupted expression of Pax-2, c-ret and c-ros involved in diabetic embrypopathy [13], genes known to modulate metanephric development [13]. Yet the signaling mechanism of hyperglycemia in impairing fetal renal morphogenesis remains to be clarified.

To identify genes entailed in hyperglycemic-induced renal embryopathy, our microarray study found glial cell line-derived neurotrophic factor (GDNF) and early growth response 1 (EGR-1) involved in developing fetal kidneys of diabetic female mice. Evidence *in vivo* and *in vitro* indicate GDNF as a key regulator for initiation of ureteric branching [14], [15], [16]. GDNF is expressed in metanephric mesenchyme (MM) before ureteric bud (UB) induction, mediates its signal via the receptor tyrosine kinase, rearranged during transfection (*Ret*), and GDNF family coreceptor α1 (*GFRα1*), then induces UB formation and for the latter regulates ureteric branching [17].

EGR-1 was originally identified as one immediate early gene [18]. Studies recognized EGR-1-bound DNA fragments containing sequence 5′-GCGGGGG CG-3′[19]–[22]. With one or more GC-rich regulatory elements (GCEs) incorporated into minimal promoter reporter constructs, EGR-1 activates transcription, demonstrating transcriptional regulatory potential [23]. Previous studies suggest a role for EGR-1 in regulation of cell division, differentiation and apoptosis [23], as well as glucose heightening EGR-1 expression [24]. One hallmark of nephrogenesis is widespread histogenetic cell death relatively late in maturation via apoptosis (genetically controlled response for cell suicide), in which many genes seem involved.

There is a growing consensus that a high-glucose milieu promote GDNF/EGR-1 signaling pathway have a functional impact on diabetic embryopathy [23], [24]. However, the underlying mechanism of GDNF/EGR-1 signaling in pathway tubulogenesis is not well understood. Given these data, we hypothesize that intraurterine under hyperglycemic environment promoting GDNF-MAPK/EGR-1 signaling is intimately involved in suppressing renal epithelial cell migration and inducing apoptosis in nascent tip of ureteric bud, ultimately leading to renal embryopathy.

## Results

### Genome-wide identification of full-length cDNA involved in hyperglycemia induced renal embryopathy

**Figure 1:**
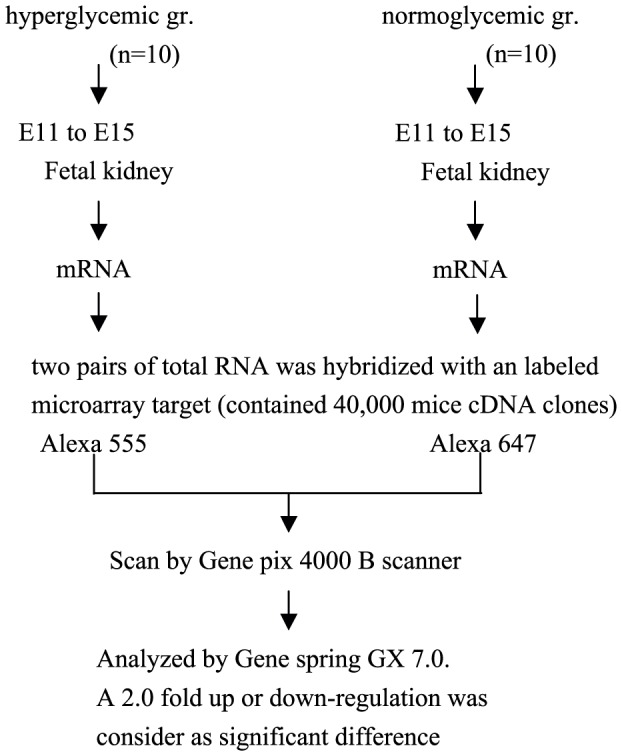
Schematic diagram of gene screening involved in hyperglycemia induce fetal nephropathy. We isolated two pairs of mRNA from E11 to E15 of fetal renal tissue either in hyperglycemic or normoglycemic state, then hybridized with 40,000 labeled cDNA microarray target. Differential expression more than twofold was defined as a positive gene (group: gr.).

To pinpoint genes involved in hyperglycemia induced renal embryopathy, we screened 40,000 labelled cDNA targets of microarray. Figure 1 plots schematic diagram of interesting gene screening, with differential expression above two-fold defined as a positive gene. We clustered redundant genes to identify six interesting ones that activate GDNF/MAPK/EGR-1 pathway. Among them, EGR-1 has been demonstrated as a regulatory protein for GDNF and essential for tubulogenesis in hyperglycemia-induced renal embryopathy.

### Validation of identified genes in hyperglycemia induced renal embryopathy

**Figure 2:**
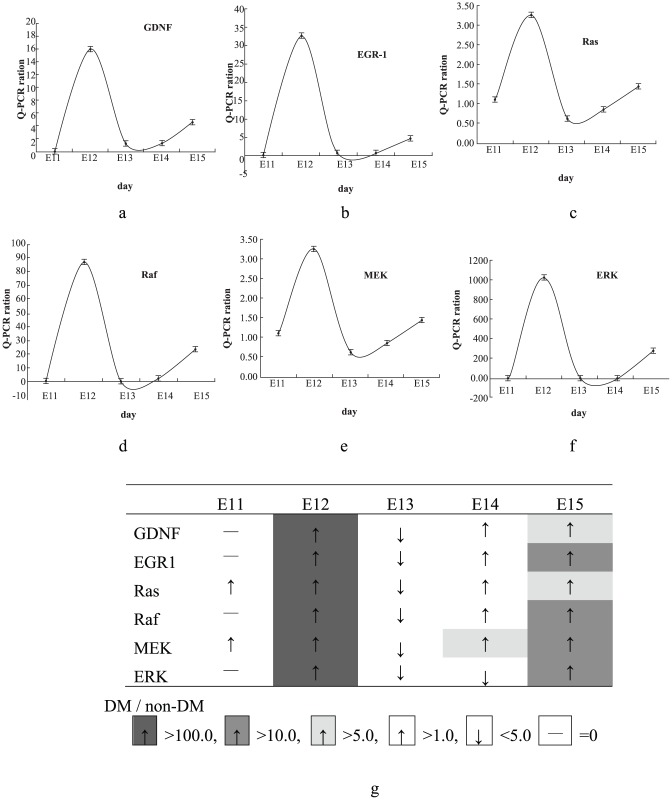
Serial gene expression patterns from E11 to E15 of fetal renal tissue. Measurement of serial GDNF, EGR-1, Ras, Raf, MEK and ERK gene expression by quantitative renal time PCR (qPCR) on cDNA from E11 to E15 of fetal renal tissue, either in hyperglycemic or normoglycemic state. Change pattern was very similar in these genes, all of them highly expressed at E12, dramatically dropping at E13, then progressively increasing from E14 to E15. (Fig. 2a. GDNF, 2b. EGR-1, 2c. Ras, 2d. Raf, 2e. MEK, 2f. ERK, and summary 2 g; n = 10).

To confirm GDNF, EGR-1, Ras, cRaf, MEK and ERK mRNA expression differing significantly between hyperglycemic and normoglycemic fetal kidney tissues observed using microarray analysis, we performed quantitative RT-PCR and compared expression patterns, as shown in Figs.2a, b, c, d, e, f, g. We found that GDNF, EGR-1, Ras, cRaf, MEK and ERK gene expression differed between hyperglycemic and normoglycemic fetal kidney tissue. Change patterns were similar in these genes, all highly expressed at E12 and dramatically dropping at E13, then progressively rising in E14–E15, (Figs.1a, b, c, d, e, f). Figure 2c shows Ras differently expressed: it reached at least 3.2 fold elevated ratio in hyperglycemic fetal kidney tissues compared to normoglycemic ones at E12 in each of ten samples (p<0.01), decreasing rapidly at E13 and progressively returning to E11 range at E15.

We further examined mRNA expression patterns of cRaf, MEK and ERK. Expression of cRaf, MEK and ERK genes was elevated in hyperglycemic fetal kidney tissues about 85, 95 and 1020 fold, respectively, higher than in normoglycemic kidneys at E12. The ratio dropped rapidly at E13 and returned or rose at E15 (Figs.1c, d, e, f). Serial pattern of mRNA expression by real-time PCR in Ras, cRaf, MEK and ERK mRNA strongly correlated with microarray analysis. Correlation coefficients in Ras, cRaf, MEK and ERK between gene expression level measured by microarray and real-time PCR were 0.791, 0.804, 0.815, and 0.823, respectively. All these showed strong positive correlation with real-time PCR-determined expression (p<0.01). Results indicate GDNF, ERG-1, Ras, cRaf, MEK and ERK mRNA suppressed during tubulogenesis in hyperglycemic fetal renal tissues.

### GDNF promotes MAPK signaling in HRPTE cells

**Figure 3:**
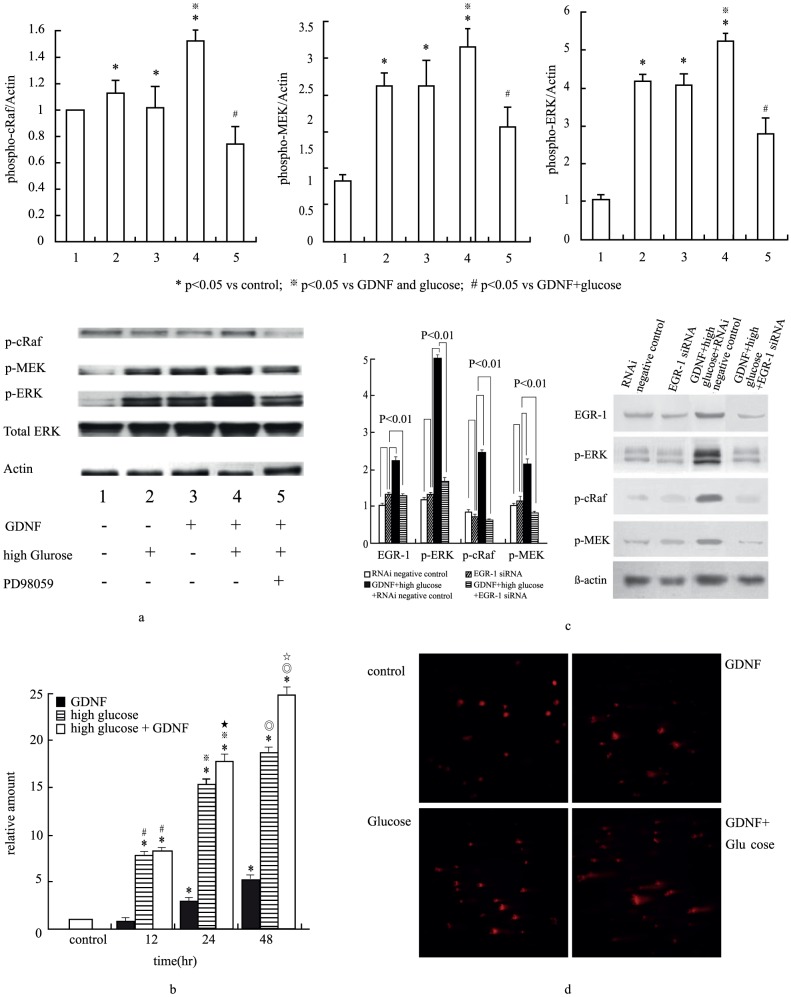
Effects of high glucose on GDNF-dependent phosphorylation of cRaf, MEK and ERK protein expression. HRPTE cells were treated with or without 20 mM glucose and/or GDNF (100 ng/ml) for 30 minutes, protein measured by Western blot. Phosphorylated cRaf, MEK and ERK significantly increased after co-treatment with high glucose and GDNF, the latter downregulated by pretreated with ERK inhibitor, PD98059 for 1 hour. Bands were quantified by densitometry, values normalized to that of β-actin protein in the same sample. Similar results emerged from three independent experiments. * vs lane 1 p<0.05, □ vs lane 2 p<0.05, # vs lane 4 p<0.05 (Fig 3a). EGR-1 mRNA expression was significantly increased in a time-dependent manner. Real-time quantitative polymerase chain reaction of EGR-1 mRNA expression in HRPTE cells, with time-dependent manner was noted in high glucose concentration (20 nM) with GDNF (100 ng/ml) for 12, 24 and 48 hours. Relative amount was compared with their own β-actin mRNA. Each point represents mean ± SD of three independent experiments. *p<0.05 vs control, #p<0.05 vs GDNF 12 hrs, □p<0.05 vs GDNF 24 hours, ★p<0.05 vs. high glucose 24 hrs, ⊚p<0.05 vs. GDNF 48 hrs, ☆p<0.05 vs. high glucose 48 hr.(Fig.3b). siRNA of EGR-1 downregulated high glucose and GDNF mediated cRaf/MEK/ERK phosphorylation.(Fig.3c). High glucose enhanced GDNF-induced DNA damage was determined by the comet assay. Higher concentration of glucose led to more damaged cells being stained (Fig.3d)

First we proved HRPTE cells can express EGR-1 by GDNF stimulation (data not shown). To ascertain the signal-transduction pathway induced by GDNF in HRPTE cells, we detected GDNF-dependent phosphorylation of MAPK signaling components by Western blot. Within the MAPK signaling cascade, MAPK kinase (MEK)-1 is a key protein that mediates signaling cascade from MAPK kinase kinase (MAPKKK) to both components of MAPK, ERK-1 and -2. Activation of ERK facilitates its translocation into nuclei, where it phosphorylates transcription activators like cRaf, MEK and ERK, all activated by GDNF (Fig. 3a, third line). Serine phosphorylation of cRaf, MEK, as well as tyrosine-threonine phosphorylation of ERK, was induced within 30 minutes, indicating the GDNF signal in cultured HRPTE cells as mediated by GDNF-MAPK signal pathways.

### High glucose concentration promotes GDNF-dependent phosphorylation of intracellular signaling in HRPTE cells

We investigated whether extracellular high glucose concentration would regulate GDNF-dependent kinase activation. Treatment of HRPTE cells with high glucose concentration (20 mM) and GDNF (100 ng/ml) resulted in enhanced GDNF-dependent phosphorylation of cRaf, MEK and ERK. It was interesting to note that serine phosphorylation of cRaf was abolished by pretreating cells with protein-tyrosine kinase inhibitor (PD98059); tyrosine-threorine phosphorylation of MEK and ERK was also down-regulated by the same protein-tyrosine inhibitor (PD98059) (Fig.3a). This implies activation of cRaf-ERK pathway representing upstream regulation. High glucose concentration caused upstream inhibition (Fig.3a). Treatment of HRPTE cells with PD98059, ERK inhibitor significantly inhibited GDNF-dependent phosphorylation of cRaf, indicating that GDNF plus high glucose concentration activates ERK-signaling through Ras.

### Hyperglycemia suppresses GDNF-induced migration of HRPTE cells

Renal epithelial cell migration is thought to play a pivotal role in fetal renal tubulogenesis. Subsequently, we examined whether hyperglycemia suppressed GDNF-induced epithelial cell migration. HRPTE cells were cultured in high glucose concentration and migration activity measured. Results showed that in control medium 15.2±3.4% of the gap was filled after 24 hours, as opposed to 35.2±5.4% in the presence of GDNF alone (100 ng/ml, p<0.001). This GDNF-induced HRPTE cell migration was suppressed by high concentration (20 mM) of glucose (GDNF vs. GDNF plus glucose: 35.2±5.4 vs. 22.6±3.8%, p<0.001).

### GDNF enhances EGR-1 mRNA expression in HRPTE cells

During hyperglycemic status, transcription EGR-1 was previously identified as a negative growth factor stimulating apoptosis. To determine whether EGR-1 mRNA expression was up-regulated by hyperglycemia, we exposed HRPTE cells to GDNF in high glucose concentration. Conceivably, EGR-1 mRNA expression was significantly increased in a time-dependent manner (Fig.3b).

### siRNA of EGR-1 abolishes hyperglycemia and GDNF mediated cRaf/MEK/ERK phosphorylation

We further analyzed EGR-1 contribution to hyperglycemia down-regulating GDNF-induced migration to HRPTE cells and GDNF-dependent phosphorylation of cRaf, MEK and ERK by small interfering RNA (siRNA) of EGR-1-mediated experiments. EGR-1 knockdown abolished high glucose concentration-suppressed GDNF-induced migration of HRPTE cells (with vs. without siRNA: 35.6±4.3 vs. 21.4±3.2% P<0.001, controls: 35.2±4.6%). Figure 3c shows EGR-1 siRNA reducing GDNF/EGR-1-induced cRaf/MEK/ERK phosphorylation by 80%.

### Hyperglycemia enhance GDNF induced apoptosis in HRPTE cells

We tested effect of hyperglycemia on HRPTE cells cultured for 48 hours in the presence of 5, 10, 15 or 20 mM concentration of glucose and in glucose-free, 1% FCS medium. Results showed GDNF-induced apoptosis in HRPTE cells (GDNF vs. control: 11.25±1.35 vs 4.95±0.82% p<0.05). We next determined whether hyperglycemia enhances GDNF-induced apoptosis, proving GDNF-induced apoptosis as significantly increased by hyperglycemia (glucose 5,10,15 and 20 mM vs. control: 5.12±0.75, 5.64±0.68, 6.74±0.82 and 7.82±1.25 vs. 4.95±0.82% p>0.01 in 20 mM glucose; GDNF 100 ng/ml plus glucose 5,10,15 and 20 mM vs. GDNF 100 ng/ml: 12.42±1.47, 14.56±1.65, 17.24±1.84 and 20.85±2.14 vs. 11.25±1.35% p<0.01 in 20 mM glucose. Each datum represents mean ± SD of six independent experiments. To assess the role of increased osmolarity in hyperglycemic culture medium, mannitol was osmolar control. Increasing HRPTE cell apoptosis was secondary to hyperglycemia, not hyperosmolarity, as addition of 15 mM mannitol did not significantly increase apoptosis compared with 20 mM glucose (data not shown). Hyperglycemia enhanced, GDNF-induced DNA damage was determined by comet assay (Fig.3d). Higher concentration of glucose led to more damaged cells being stained.

### Histological studies of hyperglycemia enhance cell apoptosis in developing fetal kidney

**Figure 4:**
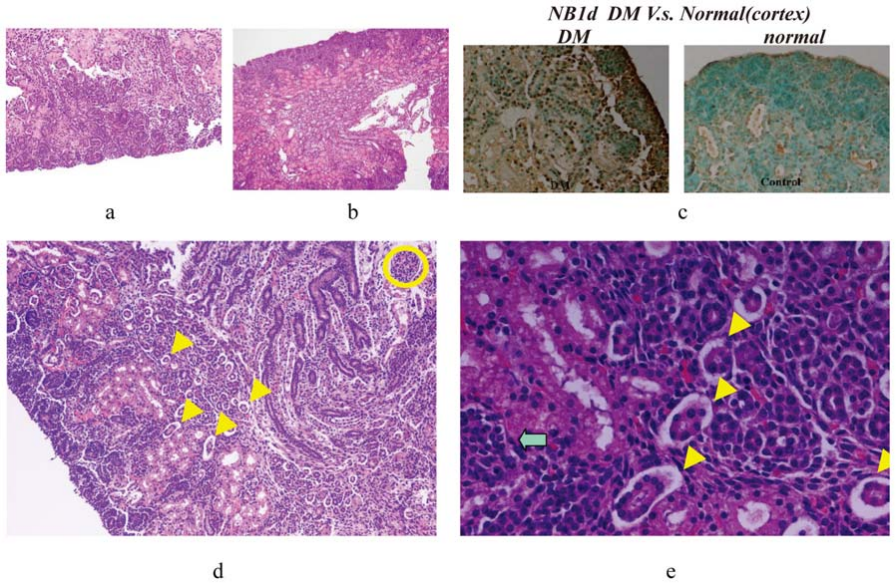
DM newborn (Day 1) mouse kidney showed nephrogenesis period between 3rd and 4th stages, revealing less demarcated cortical labyrinth and medullary rays, as well as decreased fetal glomeruli and convoluted tubules in deeper cortical and juxtamedullary regions with scattered ‘-shaped’bodies, residual advancing ampullae of ureteric buds, diminished branching of developing convolution (100×) (**Fig.4a**). Normal newborn (Day 1) mouse kidney showed nephrogenesis period near 4th (last) stage, plus clearly discernible cortical labyrinth and medullary rays with fetal glomeruli and well-developed tubules in the deeper cortical and juxtamedullary regions, developing glomeruli (100×)(Fig.4b). TUNEL assay revealed apoptosis cells increasing in metanephric mesenchyme (dark brown dots) and collapsed nephron region in newborn kidneys of the hyperglycemic group (200×) (Fig.4c). With maternal diabetes circumstances, large amounts of nephrons collapsed (HE stain, Fig. 4d 200× arrow, Fig. 4e 600× arrow). Day 1 hyperglycemic mice showed detachment between ureteric branch and metanephros (blue arrow), as well as collapse of nephrons (yellow arrow) (Figs.4d–4e).Serial changes of GDNF, EGR-1 and ERK-2 expression on branching morphogenesis: Upper parts were fetal kidney tissue from hyperglycemic versus lower parts from normoglycemic mothers. At E12, week expression of EGR-1, GDNF and ERK-2 in intermediate mesoderm of fetal kidney tissue from hyperglycemic mothers (Figs.3f, 3h and 3j). Ureteric buds unable to invade metanephric mesenchyme correlated with reduced immunoreactivity in the tubular cell basement membrane of fetal kidney tissue from hyperglycemic mother on E15 (Figs. 3g, 3i and 3k, 600×; similar results noted in six independent experiments) (Figs.4f–k).

We explored effect of hyperglycemia on tubulogenesis in developing fetal renal tissue. Comparing normal newborn (Day 1) mouse kidney to hyperglycemic mouse kidney, histological analysis found newborn kidneys of the diabetic group showing nephrogenesis between 3rd and 4^th^ period, revealing less demarcated cortical labyrinth and medullary rays, and decreased number of fetal glomeruli and developed convoluted tubules in the deeper cortical and juxtramedullary regions with scattered ‘-shaped’bodies, residual advancing ampullae of ureteric buds, diminished branching of developing convoluted tubules, and presence of adjacent metanephric mesenchyme in subcapsular superficial cortex or cortical labyrinth suggestive of abnormal nephrogenesis in both quantity and quality (Fig. 4a). Careful counting revealed newborn nephron number in offspring of diabetic mothers as significantly lower than in normoglycemic mothers (hyperglycemic vs normoglycemic newborn: 1974±131 vs 2985±124 in number [both n = 6; p<0.001]).

### TUNEL assay of kidney tissues

BrdU assay of embryonic kidneys showed no significant difference in cell proliferation between diabetic and control groups (data not shown). Still, remarkable apoptotic cells seem to increase in the collapsed nephron region (Table 1, Fig. 4c) or with decrease and collapse of nephrons (Figs.4d–4e) in fetal and newborn kidneys of the diabetic group (Table 1, Fig.4c). A serial section from the same embryo stained with anti-laminin antibody revealed that the basement membrane of the ureteric bud remained intact, indicating that ureteric bud failed to contact and invade the metanephric mesenchyme (data not shown). No matter what gestational age subgroup, p-value between diabetic and control groups was below 0.01.In addition, it was interesting that later stages of affected embryos manifested more severe cell apoptosis in the developing fetal kidney.

**Table 1:** Percentage of cell apoptosis in fetal and neonatal kidney between hyperglycemic and normoglycemic groups.

		Study group from			
		hyperglycemic mother (n = 6)		normoglycemic mother (n = 6)	
			10.5±1.4 * #		1.8±0.2
E16			12.1±2.6 * #		2.0±0.4
E17			13.5±3.44 * #		2.4±0.5
E18			37.1±9.8 #		4.4±1.6
NBD1			41.2±3.5 #		3.9±1.4

n = number of kidneys, mean ± SD, P<0.01 compared with normoglycemic group,

* Comparison to NBD1, p<0.01

# Comparison to normoglycemic mother, p<0.01

### Serial change of ERK2 and EGR-1 proteins in branching morphogenesis of renal tubule

To ferret out early changes in renal organigensis, embryos were examined at various embryonic stages. In normal embryos of mice, ureteric buds contacted metanephric rudiments at E11.5 and subsequently invaded the latter. In hyperglycemia embryos, some ureteric buds failed to invade metanephric mesenchyme. In Day 1 hyperglycemic newborns, detachment between ureteric branch and metanephros (Fig.4d), Decreasing number of glomeruli with collapse of nephrons appeared in kidneys (Fig.4d), while differentiation of metanephric mesenchymal cells was poor. (Fig.4e). Taken together, results indicate the essential process in hyperglycemic environment as defective ureteric buds to invade the metanephric radiment at around E11. When the invasion is defective, functional nephron formation may decrease and result in nephron collapse after E13.

Immunofluorescent localization of EGR-1, GDNF and ERK-2 proteins were serially observed from E12 to E15 by confocal microscopy. Serial changes of ERK2 and EGR-1 expression on branching morphogenesis at E12 and E15 were studied. Upper parts were fetal kidney tissue from diabetic mothers, lower parts were fetal tissue from normoglycemic mothers. By comparing fluorescent intensity in fetal kidneys of hyperglycemic and normoglycemic mothers, we observed E12 weaker expression of EGR-1, GDNF and ERK-2 in the intermediate mesoderm of fetal kidney tissue from hyperglycemic mothers (Figs. 4f, 4h and 4j). Ureteric buds unable to invade metanephric mesenchyme linked with reducing immunoreactivity in tubular cell basement membrane of fetal kidney tissue from hyperglycemic mothers on E15. (Figs.3g, 3i and 3k 600×). These observations may correspond to histological findings.

## Discussion

Kidney organogenesis proceeds in stages [11], [12]. Step 1 is formation of metanephric mesenchyme in the intermediate mesoderm and subsequent outgrowth of ureteric buds from the Wollfian duct. Step 2 is invasion by these buds into the metanephric mesenchyme, with several genes involved. GDNF in mice disturbs the invasion and induces renal agenesis of variable manifestation [16] Our *in vivo* study found that under hyperglycemic condition, ureteric bud invasion of metanephric mesenchyme is defective. We set out to delineate the role of maternal diabetes in modulating renal morphogenesis in fetus and to evaluate underlying mechanisms.

From serial study of microarray, we tried to identify gene targets altered from an *in vivo* model of diabetic renal embryopathy. Our study employed a combination of approaches: e.g., RNA microarray, quantitative PCR validation in a larger cohort, confirmatory experiment. We initially sought to demonstrate changes in diabetic renal embryopathy as due to change in gene expression, not post-translational degradation of protein product. We found a group of genes involving GDNF-EGR-1 pathway up-regulated on E12 then down-regulated on E13 and progressively up-regulated in hyperglycemic renal tissue, results identical to real-time PCR. In addition, immunofluorescent localization of these proteins was also observed by confocal microscopy. To understand the mechanism of hyperglycemia on GDNF-EGR-1 pathway further, our *in vitro* study first proved HRPTE that cells can express GFR-1 mRNA after stimulation with GDNF. Second, we demonstrated that high concentration of glucose induced GDNF-dependent phosphorylation of cRaf, MEK and ERK in HRPTE cells.

Functional study demonstrated GDNF-induced HRPTE cell migration was suppressed in high glucose condition. We also found that high glucose concentration can enhance GDNF via EGR-1 induced epithelial cell apoptosis. Furthermore, knockdown of ERG-1 by siRNA abolished hyperglycemia suppressed GDNF-induced migration of HRPTE cells. EGR-1 siRNA also reduced 70% of GDNF/EGR-1-induced cRaf/MEK/ERK phosphorylation. *In vivo* TUNEL assay revealed apparent increased apoptosis in newborn renal tissue of the diabetic group, corresponding to the histological study. Our data indicate that a hyperglycemic environment in utero reduces kidney size, suppress tubular cell migration and trigger apoptosis via GDNF/EGR1 signaling.

The collecting duct system of the kidney derives from the ureteric bud, and initially unbranched outgrowth of the Wolffian duct [25], [26]. The ureteric bud develops in response to GDNF, which is secreted by the nearby metanephrogenic mesenchyme; then GDNF-soaked beads elicit supernumerary ureteric buds [15], [17], [27], [28]. Once inside the metanephrogenic mesenchyme, the ureteric bud begins to form the collecting duct tree. GDNF is still required for this process [17], [25], [29]. It is reasonable to assume that signals from GDNF are passed, via GDNF receptor, to cytoplasmic signal transduction networks in the ureteric bud cells for processing and actual morphogenesis [15], [28]. Some evidence shows branching morphogenesis requiring the MAP kinase pathway [28], [29], e.g., model based on culture of Madia-Darby canine kidney cells in collagen gels, with MAP kinase pathway is required for tululogenesis [29]. In murine salivary glands, ERK 1 and ERK 2 MAP kinase are also essential for branching morphogenesis [30]. In branching morphogenesis both proliferation and apoptosis are required [19], [22]. Since discovery of EGR-1 as ‘arly growth response gene,’research was directed towards a growth and proliferation as function of the gene. However, EGR-1 expression has been linked to cell death [33], [34]. It has been shown that expression of EGR-1 either in differentiation of neuronal cells [31] or in developing animals [32]. Our study has demonstrated EGR-1 gene involvement in the apoptosis induced by hyperglycemic status in renal epithelial cells. Furthermore, enforced expression of EGR-1 in renal epithelial cell suppresses cell migration and induces apoptosis in the presence of high glucose, suggesting that this gene could participate in the decision to commit to the death pathway directly, rather than having an impact on manifestation of death process once the pathway has been activated. Our data lent *in vitro* evidence that, under high glucose condition, GDNF enhances EGR-1 mRNA expression in HRPTE cells. Hyperglycemic status suppresses GDNF-induced cell migration and enhances GDNF induced apoptosis in HRPTE cells. TUNEL assay *in vivo* revealed apparent increased cell apoptosis and collapse of nephrons in fetal and newborn kidneys of the hyperglycemic group, and confocal morphological observation demonstrated ureteric bud as unable to invade metanephric mesenchyme associated with enhanced GDNF, EGR-1 and ERK-2 expression on E15 in hyperglycemia-induced fetal nephropathy. Increased EGR-1 gene transcription in high glucose condition would thus impair the ureteric bud forming the collecting duct tree or stimuli that produce trees in branching morphogenesis then induce apoptosis and prevent ureteric bud invasion of metanephric mesenchyme. Thereafter nephron formation may be altered, resulting in nephron decrease and collapse. Based on our observations and those of others, we propose that high glucose enhances GDNF-EGR1 pathway-induced cell apoptosis, triggering nephron collapse in diabetic offspring. Further study to restore EGR-1 overexpression may protect against diabetic kidney embryopathy induced by a hyperglycemic mother.

Recent studies showed EGR-1 not only associated with cell proliferation and differentiation [18] but also contributing to pathogenesis of atherosclerotic lesion [35]. Rukhsana [36] reported that glucose-induced EGR-1 expression was mediated by PKC activation. Glucose-induced expression of EGR-1 represents an early step in the development of diabetes-related cardiovascular complications [24]. Cross-talk between EGR-1 dependent transcription and various intracellular signaling cascades need be identified in future.

Several diabetic animal models, including STZ-induced diabetic rats [37], STZ-induced diabetic mice [38], diabetic Chinese hamsters [39], and the OLETF rat model of Type 2 diabetes [8] have been established. The STZ-induced mice model was commonly used in the study of diabetic embryopathy. STZ is an unstable product with a biologic half-life in cell culture medicine. Since STZ administration does not always induce diabetes, we had the opportunity to examine nephrogenesis in fetuses of STZ-exposed mice with or without diabetes. From *in vivo* study using Nephron-Cyan Fluorescence Protein-Transgenic mice, they found that fetal renal damage depends on level of maternal hyperglycemia but not STZ administration or length of exposure to STZ [40]. The present study demonstrates that high glucose levels enhanced apoptosis of renal tubular epithelial cells both *in vivo* and *in vitro*. We believe it unlikely that a small amount of STZ exerts toxicity *in utero* in our model.

In conclusion, this study implicates molecules of GDNF/EGR-1/MAPK pathway that appear involved in development of diabetic renal embryopathy. This novo finding is particularly important because it implicates EGR-1 as a regulator involved in crucial cellular processes in hyperglycemic state: e.g., apoptosis regulation. Results suggest EGR-1 level playing a vital role in renal tubular epithelial cell migration and survival during tubular morphogenesis in hyperglycemic condition.

## Materials and Methods

### Animals

C57BL/6J mice, 6 to 8 weeks old, were purchased from National Laboratory Animal Center (NLAC), Taiwan, and maintained on a 12-h alternating light/dark cycle and supplied with commercial pelleted diet (NLAC). All mice were housed and in treated accordance with policy of the Laboratory Animal Care and Use Committees, and the animal protocol was approved by Changhua Christian Hospital. For the induction of diabetes, females were injected intraperitonealy with 50 mg/kg STZ (Sigma, St. Louis, MO) for 3 consecutive days [38]. One week later after treatment, a blood sample was obtained from the tail vein, and plasma glucose levels were determined using a glucose analyzer (Advantage II; Roche Diagnostics GmbH, Mannheim, Germany). Mice with values >300 mg/dl were considered manifestly diabetic and were included into the experimental group. Females were injected with 100 mmol/L of citrate buffer only and served as non-diabetic normal controls.

### Embryonic renal tissue RNA preparation

Embryos were dissected on gestational Days 12(E12), 13(E13), 14(E14), 15(E15), and newborn Day 1 (NBD1). Right kidney was removed and fixed in 10% formalin at 4°C overnight, tissues dehydrated and embedded in paraffin, then sectioned at 4 µm for hematoxylin-eosin (H-E) staining, proliferation, apoptosis study, as well as fluorescence *in situ* hybridization (FISH). Left kidney was removed for RNA extraction.

### Construction of full-length cDNA library and arranged cDNA pool

Total RNA isolated from normal and hyperglycemia embryo by (Trizol method. After a crude selection, 1) 28 s rRNA has a relatively stronger signal to 18 s rRNA in RNA gel evaluation 260/280 and 260/230 nm OD ratio is higher than 1.8 by OD detection, three pairs of normal/hyperglycemia total RNA were selected to a next step of microarray quality control test. A similar total RNA evaluation Bioanalyzer with higher sensitivity detected total RNA quality before microarray. Evaluation indexes are (1) 28 s/18 s rRNA ratio above 1.5; (2) RNA integrity number (RIN) higher than 8.0; (3) 40% or higher summation of 28 s rRNA and 18 s rRNA. Afterwards, two pairs of total RNA were qualified to be recombinant as labeled cDNA target. We use an indirect labeling method to improve sensitivity and strength of cDNA target signals when microarray slides are scanned, target cDNAs labeled with two Alexa dyes Alexa555 and Alexa647 individually (Fig.1). Expression difference of two between normal and hyperglycemic embryos determined which genes were entered.

### GeneSpring analysis

To avoid different amounts of cDNA target interfering with further analysis of gene expression, equal amounts of cDNA targets were separated into Pairs 1 and 2, each pair hybridized with an individual microarray slide scanned by GenePix 4000B scanner and analyzed by Genespring GX 7.0 (Agilent Technologies, Palo Alto, CA) with defined algorithm to filter out differentially expressed genes that had marginal flags in at least four of seven samples (Fig.1). Differentially expressed genes showing at least two-fold change were selected, further correlated, then pooled with cluster analysis and gene ontology (GO) classifications.

### Real-time PCR with SYBR green assay

Total RNA was isolated from prepared embryonic left kidney using TRI reagent (Gibco BRL, Grand Island, NY) and treated with RNase-free DNase (Gibco BRL). RT-PCR was performed using Purescript RNA Purification System (Gentra, Minneapolis, MN). Each PCR cycle was 94°C for 30 seconds, 62°C for 30 seconds, and 72°C for 1 minute, 30 cycles used to assay GDNF and EGR-1. Five µl of cDNA (1–10 ng) was mixed with SYBR green PCR core reagent or SYBR green PCR master mix reagent. Thermal cycling conditions were determined according to ‘hermal cycling parameters for primer optimization’rules and CT value of an unknown sample compared with endogenous control for quantification of gene expression. Primers were designed using to Primer Express Primer Design software, as shown below in Table 2.

**Table 2:** Primers of real-time PCR.

GAPDH	F. GGGTGTGAACCACGAGAAAT
	R. CCTTCCACAATGCCAAAGTT
GDNF	F. GTTATGGGATGTCGTGGCTGTC
	R. CCGTTTAGCGGAATGCTTTCTTAG
EGR-1	F. GGGAGCCGAGCGAACAA
	R. CAGCGCCTTCTCGTTATTCAG
ERK2	F. TCTCCCGACCAAAAATAAGG
	R.AGAAGTCAGAGGCAGGTGGA
ERK1	F. TCCTTTTGGATCTGGTCCTG
	R. CCCCAGCAAAGTGAGAGAAG
MEK1	F. GTGAACTCACGTGGGGAGAT
	R. GGCGACATGTAGGATCTCGT
MEK2	F. CCTACATCGTGGGCTTCTA
	R. CCTTCCCCAAGATGTCTTCA
MKP	F. ACTGTCCGGATCTGTGCTCT
	R. ACCACCCTGGTCATAGATCG
Raf 1	F. TTGTTTCCCCAGATCCTGTC
	R. CTGGTAGCCTTGGGGATGTA

### Western blot

Embryonic renal tissue were resuspended in lysis buffer (1% NP-40, 0.1% SDS, 0.5% sodium deoxycholate, 150 mM NaCl pH8, 1 mM PMSF, and protease inhibitors). A total of 100 µg of protein was separated by 12% SDS-PAGE and transferred onto PVDF membranes. Blots were blocked with 5% non-fat milk in TBS + 0.05% Tween 20 (TBST). Primary antibodies were anti-cRaf, anti-ERK and MEK mAb (1/1000) (Cell Signaline Technology, Beverly, MA), and anti-β actin mAb (Sigma) [41].

### Immunohistochemistry

Immunohistochemical staining was performed by immunoperoxidase method previously described [36].

### Proliferation study (BrdU assay)

We used 5-Bromo-2′-deoxy-uridine Labeling and Detection Kit I (Roche Diagnostics GmbH, Penzberg, Germany) by immunoflurescent technique to determine proliferation of kidney samples. Pregnant female mice were sacrificed 1 our after injection of BrdU labeling reagent (1 ml/100 gm of body weight). Prepared sections were covered with a sufficient amount of anti-BrdU working solution after dewaxing and then incubated for 30 minutes at 37°C [38]. Finally, sections were washed twice again prior to fluorescent microscopic evaluation (Olympus BX51TRF microscope) [42].

### Apoptosis study (TUNEL assay)

Fragmented DNA was labeled by reaction mixture, according to manufacturer' instructions, bound probes detected with 3, 3′-diaminiobenzidine (DAB) as substrate (TdT-FragEL^TM^ DNA Fragmentation Detection Kit, Calbiochem, Germany) [42].

### Quantitative analysis

Immunostaining for BrdU and TUNEL assay was quantified by previously published methods [41], [43]. We counted nuclear BrdU or TUNEL stains as percentage of positive cellular staining.

### Cell culture

Human renal proximal tubule epithelial cells (HRPTE cells) were purchased from ATCC (American Type Culture, Collection, USA). HRPTE cells were grown in 1 to 1 Dulbecco' modified eagle medium (Gibco, USA) and F-12 nutrient mixture (Gibco, USA) plus 10% fetal bovine serum (FBS, Gibco Lab, Grand Island, NY), 1% sodium pyruvate, 1% non-essential amino acid, 2 mM L-glutamine, 5 µg/ml transferin, 1% Penicilln 100 U/ml and 10 mg/ml streptomycin.

### Cell viability and apoptosis assays

To assess cell viability, aliquots of cells were mixed with Trypan blue (1∶1) and loaded on to a haemocytometer; and percentage of dead cells per sample calculated, with at least three survival assays carried out independently. Apoptosis was assayed using propidium iodide (PI) staining and FACS analysis. Data were presented using the dot density plot function. Data were analyzed by Cell Quest software package (Becton Dickinson) [42], [43].

### Comet assay

Approximately 5×10^3^ cell/ml were incubated with doxorubicin or glucose (20 mM) for 48 h at 37°C, isolated, and examined for DNA damage, using the Comet assay previously described [39].

### *In vitro* GDNF-induced migration of HRPTE cells

We investigated *in vitro* GDNF-induced migration of HRPTE cells by a modified Boyden Chamber assay. Briefly, HRPTE cells were seeded onto tissue inserted containing a polyethylene terephthalate (PET) filter with 8 µm pore size (Falcon; Becton Dickimson). The medium was changed to MEM without addition in the upper well and with GDNF (100 ng/ml) or GDNF (100 ng/ml) plus glucose (20 mM) in the well. After 24 hours, cells were removed from the surface of the inserted by gentle scrubbing; cells on the bottom of the insert (which had migrated through the filter) were fixed with −20°C methanol and stained with crystal violet [44]. The surface of filters was screened for cells under a microscope (10 fields/filter), cell number counted by Image-Propus system (Media Cybametics, Version.7.0.0591, USA, 2009).

### Hyperglycemia-induced apoptosis

HRPTE cells were treated with glucose concentrations (20 mM) in the presence or absence of GDNF (100 mg/ml) in serum free/glucose-free medium and maintained in culture for 48 hours.

### siRNA transfection

One pair of small-interfering RNAs (siRNA) was synthesized by Invitrogen Life Technology (Invitrogen Taiwan, Ltd.). HRPTE cells were transfected with EGR-1 siRNA (20 µM) using Lipofectamine RNAiMAX (Invitrogen Life Technology). After 48 hours, proteins were extracted for Western blot analysis. Cellular lysates were prepared as described previously (Chiu et al., 2009), proteins resolved on SDS-PAGE and transferred to Immobilon polyvinyldifluoride (PVDF) membranes. Blots were blocked with 4% BSA for 1 h at 22.2°C and probed with rabbit anti-human Ab against cRaf, MEK, MEK (Cell Signaling Teclmology, Beverly, MA) and β-actin (Sigma) (1/1000) for 1 h at room temperature. Blots were visualized by ECL using Eastman Kodak X-OMATLS film, quantitative data were obtained by computing densitometer and Image Quant software.

### Statistical analysis

Statistical analysis was performed with commercially available SPSS 12.0 software (SPSS Inc., Chicago, IL). Continuous variables were expressed as mean ± standard deviation (SD), intergroup comparisons by Student' t-test, quantitative PCR data analyzed via non-parametric Wilcoxon paired test. Significant differences were defined as p<0.05; group comparison was by one-way ANOVA, p-value <0.05 was considered significant.
